# Case report: Multiple disconnection patterns revealed by a multi-modal analysis explained behavior after a focal frontal damage

**DOI:** 10.3389/fneur.2023.1142734

**Published:** 2023-03-17

**Authors:** Elena Monai, Erica Silvestri, Marta Bisio, Annachiara Cagnin, Marco Aiello, Diego Cecchin, Alessandra Bertoldo, Maurizio Corbetta

**Affiliations:** ^1^Clinica Neurologica, University Hospital of Padova, Padua, Italy; ^2^Department of Neuroscience, University of Padova, Padua, Italy; ^3^Department of Information Engineering, University of Padova, Padua, Italy; ^4^Padova Neuroscience Center (PNC), University of Padova, Padua, Italy; ^5^Department of Biomedical Sciences, University of Padova, Padua, Italy; ^6^IRCCS SDN, Naples, Italy; ^7^Nuclear Medicine Unit, Department of Medicine, University Hospital of Padova, Padua, Italy; ^8^Venetian Institute of Molecular Medicine (VIMM), Padua, Italy

**Keywords:** disconnection, multi-modal mapping, networks, brain lesion, behavior

## Abstract

**Introduction:**

There is overwhelming evidence that focal lesions cause structural, metabolic, functional, and electrical disconnection of regions directly and indirectly connected with the site of injury. Unfortunately, methods to study disconnection (positron emission tomography, structural and functional magnetic resonance imaging, electroencephalography) have been applied primarily in isolation without capturing their interaction. Moreover, multi-modal imaging studies applied to focal lesions are rare.

**Case report:**

We analyzed with a multi-modal approach the case of a patient presenting with borderline cognitive deficits across multiple domains and recurrent delirium. A post-surgical focal frontal lesion was evident based on the brain anatomical MRI. However, we were able to acquire also simultaneous MRI (structural and functional) and [18F]FDG using a hybrid PET/MRI scan along with EEG recordings. Despite the focality of the primary anatomical lesion, structural disconnection in the white matter bundles extended far beyond the lesion and showed a topographical match with the cortical glucose hypometabolism seen both locally and remotely, in posterior cortices. Similarly, a right frontal delta activity near/at the region of structural damage was associated with alterations of distant occipital alpha power. Moreover, functional MRI revealed even more widespread local and distant synchronization, involving also regions not affected by the structural/metabolic/electrical impairment.

**Conclusion:**

Overall, this exemplary multi-modal case study illustrates how a focal brain lesion causes a multiplicity of disconnection and functional impairments that extend beyond the borders of the anatomical irrecoverable damage. These effects were relevant to explain patient’s behavior and may be potential targets of neuro-modulation strategies.

## 1. Introduction

There is overwhelming evidence that local structural damage induces structural and functional disconnection effects remotely from the site of injury (1–4) hence directly supporting Von Monakow’s concept of diaschisis (5, 6).

The discovery of remote physiological alterations and their behavioral effects has been documented in neuroscience research over the last 40 years using several methods (7) that are available in the clinical setting. Among these, measures of glucose metabolism with positron emission tomography (PET) (8, 9); alterations of local activity and inter-regional correlation among brain regions or networks through resting-state fMRI (rs-fMRI) (2, 4, 10–12); structural disconnection (SDC) with diffusion imaging or structural connectome atlas (13–15); and finally, electrophysiological alterations with electroencephalography (EEG) (16–18). However, most investigations have been conducted using these methods in isolation or partial combination (19–21), with the result that a clear understanding of how different signals relate to each other is missing (22, 23).

An understanding of the relationship among multiple type of disconnection has wide relevance in clinical neuroscience (24–30).

Moreover, a detailed investigation at the level of single subjects represents an opportunity to improve our knowledge of structure–function relationships and an opportunity to differentiate between the irreversible anatomical damage and network-related functional impairment.

Relevantly, the latter may benefit of neuro-modulatory strategies in patients with focal lesions (31).

We report here a patient with a post-surgical focal lesion of the right medial frontal lobe and fornix after craniopharyngioma excision whose disconnection was studied with multiple brain imaging methodologies. His cognitive profile showed borderline performance across multiple cognitive domains. In addition, the patient presented recurrent delirium with VHs with worsening cognitive performance.

We acquired simultaneous structural and functional MRI and [^18^F]FDG metabolic information using a hybrid PET/MRI scan along with multiple neuropsychological evaluations (NPEs) and EEGs (obtained both in and out of the delirium episodes). This allowed us to document local and remote disconnection and metabolic effects as well as the dynamic of electrophysiological abnormalities that explained patient’s behavior.

## 2. Materials and methods

### 2.1. Case description

The patient, a 52-year-old man, underwent brain surgery *via* craniotomy for craniopharyngioma a year and a half before the study. Brain MRI after surgery showed post-surgical damage in the right frontal lobe (Supplementary Figure S1). Hormonal replacement therapy was started due to post-surgical hypopituitarism. At home, the family, and the patient himself noted problems with episodic memory. Nine months after surgery he underwent an EEG recording (EEG1). One year and a half after surgery he was admitted due to his first episode of delirium with visual hallucinations.

The patient presented with psychomotor slowing, drowsiness, spatiotemporal disorientation and the development of a psychotic state with agitation and disorganized thoughts. Two EEGs, a structural MRI and a NPE were performed during delirium, respectively at 3 (EEG2) and 12 (EEG3), 9, and 11 days after admission. There was bilateral slowing on the EEG during delirium (Supplementary Figure S2).

A systemic infection with a raise in serum inflammatory indices was detected. Cerebral spinal fluid was negative for infections and neuro-degenerative markers. He recovered from delirium after 15 days from admission, after treatment with a cycle of antibiotics and antipsychotics (risperidone). He was discharged after 28 days. At day 20 of admission, when delirium symptoms were resolved, he underwent an integrated [^18^F]FDG PET/MRI scan and, 4 days and 1 month later, repeated NPEs. A total of 14 months after the first episode, another frank episode of delirium occurred with disorientation, agitation and disorganized thoughts, and the patient underwent another EEG (EEG4) the day after symptoms’ acme (day 7 of admission). This second episode of delirium lasted for 8 days and resolved after treatment with haloperidol.

### 2.2. Neuropsychological assessment

Neuropsychological evaluations were obtained during delirium, out of delirium on day 24 after admission and at 1 month after discharge at his baseline. Patient performed a multiple domain battery consisting of memory, attention, executive functions, language, and visuo-spatial sections (Supplementary Table S1; Supplementary Figure S3).

### 2.3. Pet/MRI data details

A simultaneous hybrid [^18^F]FDG PET/MRI scan was acquired on a Siemens Biograph mMR (Siemens Healthcare, Erlangen, Germany) equipped with a PET compatible 16-channels head–neck coil.

The MR imaging protocol included: (a) a T1-weighted image (TR/TE 2400/3.2 ms, voxel 1x1x1mm^3^), (b) a T2-weighted image (TR/TE 3200/536 ms, voxel 1 mm × 1 mm × 1 mm), (c) a T2-weighted Fluid Attenuated Inversion Recovery (FLAIR, TR/TE 5000/395 ms, voxel 1 mm × 1 mm × 1 mm), and (d) 10 min of eyes-open resting state fMRI (rs-fMRI: TR/TE 1100/30 ms, voxel 3 mm × 3 mm × 3 mm, 40 slices).

PET imaging started 45 min after the [^18^F]FDG intravenous bolus injection and lasted 20 min. The PET static image (voxel size 2.8×2.8×2.0 mm^3^) was reconstructed off-line by means of the Siemens e7-tool software according to (31).

Two different datasets were used as healthy control groups. For the rs-fMRI data, we used 308 subjects (125 females; mean age 36.96 ± 18.40 years) of the publicly available MPI-Leipzig Mind-Brain–Body (LEMON) dataset (32, 33).

For the PET data set, the healthy control group (henceforth PET HC dataset) consisted of 26 subjects (16 females, age range 40–78 years) from a previous study by Aiello and colleagues (34). PET measurements started 30 min post injection and acquired for 15 min with reconstruction voxel size of 1.12 × 1.12 × 2.03 mm.

### 2.4. MRI data: Methods and analyses

The patient’s lesion was manually segmented on structural MRI scan (T1-weighted, considering also FLAIR and T2-weighted sequences) using the itk-SNAP software.^1^

The lesion mask was non-linearly mapped into the MNI152 standard space and the SDC map was calculated with BCB toolkit (14) using the default set of healthy controls. We identified the most affected white matter (WM) tracts by computing the percentage overlap between the SDC map and each anatomical tract provided by the toolbox (a full list of tracts is reported in the Supplementary Table S2) and normalizing for the volume of the tract. A tract with a volume involvement of more than 10% was considered to be severely impaired.

Functional scans underwent a state-of-the-art preprocessing as in (35). In addition, a high pass filtering (cutoff frequency 0.008 Hz) and an independent component analysis (ICA)-based denoising (36) were performed to remove further sources of noise.

The functional data were used to extract three main measures: 1) the spatial pattern and strength of the main resting state networks (RSNs); 2) their inter-network connectivity; and 3) the local activity synchronization.

To address the first two, we followed the same procedure as in Silvestri et al. (37). Overall, 45 independent components (IC) were identified as representative of intrinsic connectivity networks (or RSNs) and grouped into 10 different networks: visual (VIS), sensorimotor (SMN), auditory (AUD), cingulo-opercular (CON), dorsal-attention (DAN), frontoparietal (FPN), default mode (DMN), cognitive control (CCN), frontal (FRN) and language (LANG) network. Components were estimated at the single subject level through the group guided ICA (38). Then, modification of RSNs spatial pattern and strength were quantified using the cosine similarity (CSM) between patient’s and group’s independent component maps. Statistically significant alterations were assessed comparing the patient’s CSM value with the empirical statistical distribution of the CSM obtained in the control dataset within a permutation test framework (50,000 permutations, threshold of-2 standard deviations from the HC average CSM, significance level 0.05).

In both the patient and each HC subject, the inter-network connectivity was quantified computing the Person’s correlation between each pair of independent components (RSN) time courses. For statistical purposes, the correlation values were z-Fisher transformed. As for intrinsic connectivity: significantly hyper-or hypo-connected couple of RSNs were detected by comparing the strength of each inter-network connection with the empirical statistical distribution of this connection in the control group (50,000 permutations, threshold of ±2 standard deviations from the HC average, significance level 0.05).

Finally, we computed the regional homogeneity (ReHo) of the resting state functional signal, a measure of local activity synchronization, as introduced in (39). The ReHo measures were computed in regions of interest (ROI) of the Hammersmith anatomical atlas (40) averaging voxel-wise ReHo values within each region. With a permutation test framework, hyper-or hypo-integrated ROIs were detected as regions with ReHo values outside of the normal range of average ReHo ±2 standard deviations (50,000 permutations, significance level 0.05).

### 2.5. Pet data: Quantification and statistical analysis

Since the patient and control PET data were acquired using the same scanner but with slightly different protocols, we designed an analysis strategy less sensitive to acquisition protocols. The [^18^F]FDG standard uptake value ratio (SUVR) was computed on both dataset using the pons [as defined in the Hammersmith atlas (40)] as reference region. Next, regional changes of brain metabolism were estimated at the ROI-wise level though the metabolic laterality index (LI). As for ReHo, ROIs were defined according to the Hammersmith atlas for the gray matter. The SUVR values at the voxel level were averaged within each ROI (*i*), and a LI was computed as the difference between each left hemisphere ROI and its homologous regions in the right hemisphere normalized by the sum of the SUVR of the two regions:

$$LI_{i}=\frac{SUVR_{i,L}-SUVR_{i,R}}{SUVR_{i,L}+SUVR_{i,R}}.$$

Hence, since the lesion was in the right hemisphere, a positive LI indicates a relative hypometabolism in the damaged (right) hemisphere, as compared to the undamaged (left) hemisphere. Regions with significant hypo/hyper metabolism were identified by comparing each patient’s ROI LI with an empirical distribution of the same ROI LI in the PET HC dataset. Using a permutation test framework (50,000 permutations, threshold of ±2 standard deviations from the PET HC average, significance level 0.05).

### 2.6. Electroencephalography data: Detailed description and analyses

EEG were recorded using 21 electrodes placed according to the standard 10–20 international system.

All the sessions consisted of about 20 min of resting state activity during which the patient was asked to rest and keep his eyes closed. Raw EEG data underwent the following pre-processing in EEGLAB toolbox (41): high-pass filtering with a cut-off frequency of 0.5 Hz; low-pass filtering with a cut-off frequency of 45 Hz; re-referencing using the average signal as reference (42); ICA computation (43).

In addition, a visual inspection was carried out to mark and delete additional bad temporal epochs (44). The rest of the analysis was then carried out on post-processed clean data.

We ran a power spectral density analysis in four consecutive frequency bands: delta (1÷4 Hz), theta (4÷8 Hz), alpha (8÷13 Hz), and beta (13÷20 Hz) (45).

## 3. Results

### 3.1. Neuropsychology

At his baseline, the patient was oriented to space/person, and partially to time. The NPE highlighted borderline performance in multiple cognitive domains including memory, executive, and visuo-spatial functions (Supplementary Figure S3).

During delirium he showed severe attentive, executive, memory, and visuo-spatial deficits (Supplementary Figure S3), and visual hallucinations.

At day 25 post-admission he underwent a multi-domain NPE that showed a substantial return to baseline condition (Supplementary Table S1). A total of 14 months later, he suffered a second episode of delirium. At that time no neuropsychology was obtained.

### 3.2. Lesion, structural disconnection, and hypometabolism

**Figure 1:**
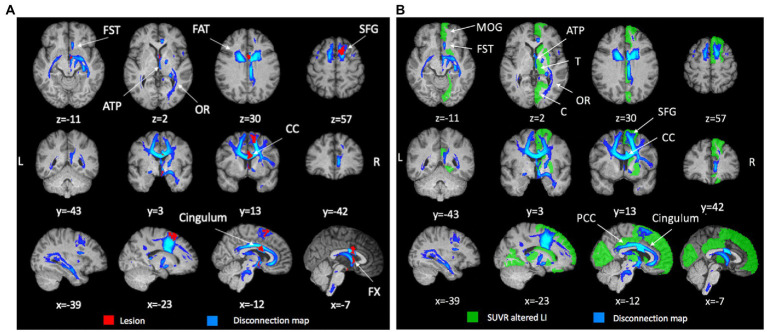
Anatomical lesion, structural disconnection map and metabolic asymmetry. **(A)** Anatomical lesion and associated structural disconnection map. T1-weighted structural MRI scan showing the anatomical lesion (red) in the midline frontal structures and the associated structural disconnection (blue) extending posteriorly and contralaterally (FST, fronto-striatal tract; ATP, anterior thalamic projection; OR, optic radiation; FAT, fronto-aslant tract; SFG, superior frontal gyrus; CC, corpus callosum). **(B)** *Structural disconnection and metabolic asymmetry*. T1-weighted structural MRI scan showing the structural disconnection (blue, threshold 20%) and regions with significative metabolic asymmetry (>2SD; green; SFG, medial orbital gyrus (MOG), caudate, putamen, thalamus (T), posterior cingulate cortex (PCC), cuneus, lingual gyrus).

The structural lesion was limited to anterior mesial region located along a track between the right posterior dorsolateral part of the superior frontal gyrus (SFG) and the hypothalamus, passing through the anterior cingulate, the anterior portion of the corpus callosum (CC), and the fornix (Figure 1A).

This lesion was associated with widespread WM tract disconnection in both anterior and posterior regions of the brain. The disconnection was predominant in the ipsilesional hemisphere with a partial contralateral extension due to the involvement of the CC, fornix and anterior commissure (Figure 1A). Among the significantly disconnected tracts there were FST (fronto-striatal tract), ATP (anterior thalamic projection), OR (optic radiation), FAT (fronto-aslant tract), SFG (see Supplementary Table S2 for all significantly disconnected tracts).

Overall, the lesioned hemisphere showed a relative hypometabolism (Supplementary Figure S4) as compared to the contralateral. The regions with a statistically significant relative hypometabolism were adjacent to the lesion like the SFG or along the medial wall like the posterior cingulate cortex (PCC). Homolateral subcortical regions like thalamus, putamen, and caudate were also affected. Finally, remote regions in the occipital cortex (lingual gyrus and cuneus) were hypometabolic (Figure 1B). When we examined the pattern of SDC vis-à-vis the map of significantly hypometabolic regions, we found a good topographic match between the WM disconnections and the relative reduced metabolism of the cortical and subcortical areas linked by the impaired bundles (Figure 1B). This match was even more evident when looking at unthresholded maps of structural disconnection (Supplementary Figure S5). Of note, regions that were bilaterally disconnected, as medial prefrontal cortices (mPFC), showed on the [^18^F]FDG PET SUVR map an hypometabolism not captured by the LI (Figure 1B; Supplementary Figure S6).

### 3.3. Alterations of functional connectivity and local synchronization

**Figure 2:**
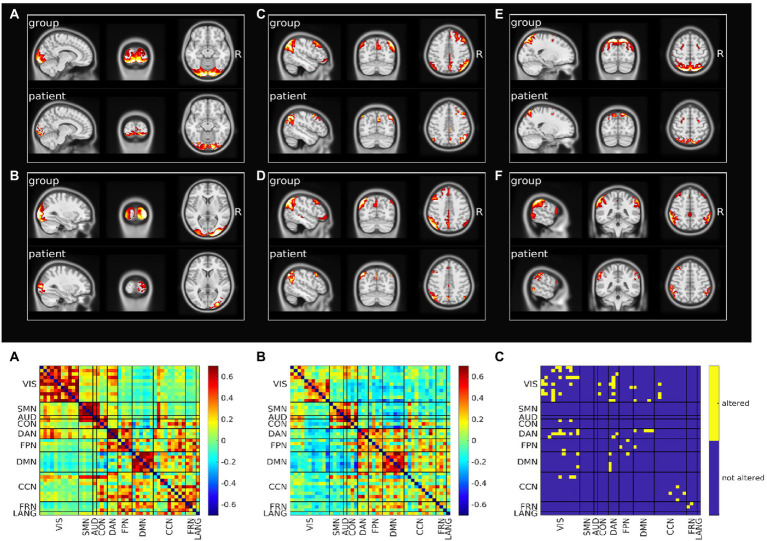
Resting state networks functional connectivity analysis. Top: Spatial pattern of representative altered resting state networks. VIS **(A,B)**, DMN **(C,D)**, DAN **(E)**, and FPN **(F)**. In each panel resting state network spatial pattern is reported for the group of healthy subjects and for the patient, respectively in the upper and lower part of the panel. Bottom: Functional connectivity between resting state networks. Average across the healthy group is shown in panel **A**, patient’s connectivity in panel **B**, and patient’s altered connections in panel **C**.

Figure 2 (top) shows representative altered components for the most five affected RSNs (VIS, DMN, DAN, FPN and CCN; Supplementary Figures S7, S8 shows all altered components).

We also analyzed the FC within-between components divided by RSN. Figure 2 (bottom) shows the FC matrix of the group of healthy controls vs. that of the patient, and the statistically significant altered connections based on a permutation test. The VIS network was the most affected in terms of number of altered connections (*n* = 13), even though visual regions were farther away from the primary lesion. Within DAN, FPN, CCN, FRN alterations also occurred. The VIS network lost connectivity with many non-sensory networks as DAN, DMN, CON, CCN and FPN. Links between DMN and DAN and DMN and FPN were additionally impaired. Of relevance, the altered connections also involved networks that were not affected in their spatial extent (Supplementary Table S3). When we compared the spatial maps of SDC, glucose metabolism and voxels showing altered FC, FC alterations showed a pattern more widespread than alterations of SDC or metabolism (Supplementary Figure S9).

The final analysis concerned the level of local activity synchronization (ReHo). In details, the following ROIs showed a decreased ReHo: SFG (bilateral), middle frontal gyrus (bilateral), precentral gyrus (bilateral), posterior temporal lobe (right), lateral part of anterior temporal lobe and middle and inferior temporal gyrus (left), inferior-lateral remainder of parietal lobe (left), superior parietal gyrus (bilateral), lateral reminder of occipital lobe (bilateral), fusiform gyrus (right), cuneus (bilateral).

**Figure 3:**
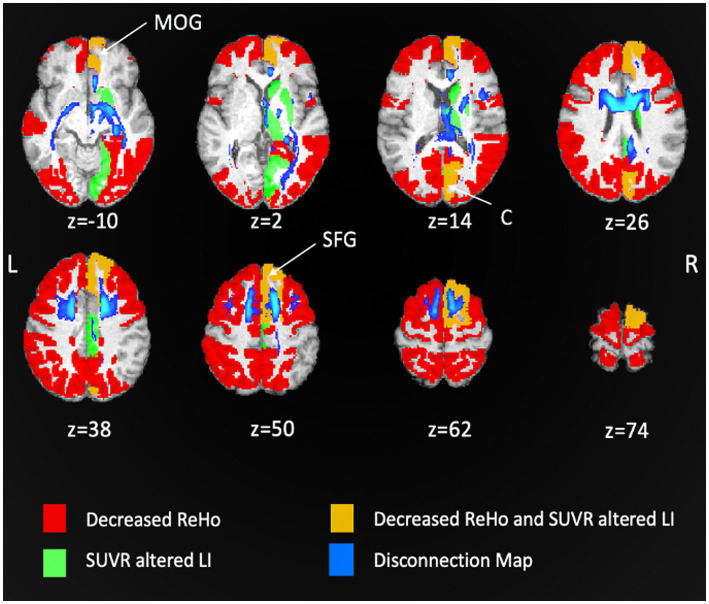
Overlap maps of decreased ReHo, [^18^F]FDG SUVR altered laterality index (LI) and disconnection map. Patient’s regions with decreased ReHo are shown in red, SUVR altered LI in light green. The overlap between decreased ReHo and SUVR altered LI is depicted in orange and corresponds to right medial prefrontal and right medial occipital cortices. The structural disconnection map is shown in blue (MOG, medial orbital gyrus; C, cuneus; SFG, superior frontal gyrus).

Figure 3 shows a voxel wise overlap map comparing ReHo abnormalities with relative hypometabolism and SDC. Note that the cortical regions showing both metabolic asymmetry (i.e., a LI different from normality) and decreased ReHo are relatively few and mainly near the lesion in prefrontal cortices. The regions showing a decrease of local synchronization are widespread and bilateral, and match those showing abnormal FC (compare Figure 3 with Supplementary Figure S9).

### 3.4. Global and local electroencephalography abnormalities

Baseline EEG showed a lower alpha peak frequency (APF) value (7.2632 Hz) compared to the standard reference (8–13 Hz) with a slight left–right alpha asymmetry in occipital regions (left>right). Delta activity was present on right frontal regions. A predominance of beta power over the right frontal regions was also observed. This pattern is consistent with the right frontal lesion causing increase delta/beta power in the right hemisphere, and a relative loss of alpha power in the right occipital lobe, with an overall lower APF.

**Figure 4:**
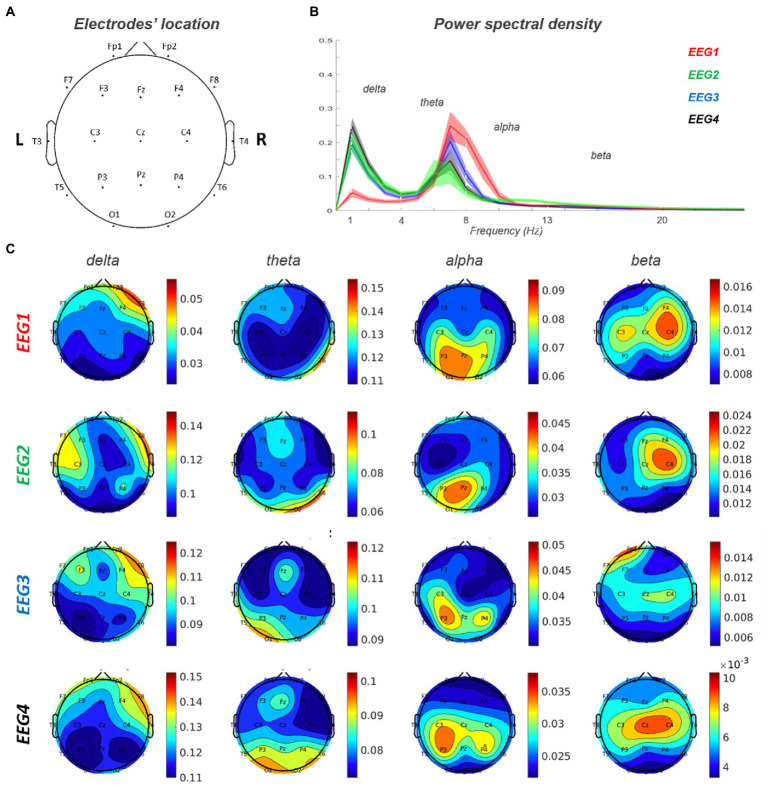
EEG 1–4 power spectra analysis. **(A)** Electrodes’ location on the scalp. **(B)** Power spectral density for each of the four EEG records. **(C)** Topoplots for each frequency band (delta, theta, alpha and beta) in EEG 1–4. EEG1 is out of delirium while EEG 2–4 are during delirium.

During the episodes of delirium (EEG2-4), at the global level, there was a general slowing of the background activity with an increase in delta activity and a reduction in the value and power of the alpha peak (Figure 4).

At the spatial level, the delta activity increased in power over right frontal region and extended contralaterally and posteriorly to centra-parietal regions. Furthermore, in correspondence of the posterior regions, there was an increase in theta and a reduction in alpha activity. Alpha activity showed a left>right asymmetry that was more evident during delirium with also a slight posterior-to-anterior shift in EEG3-4 (left>right).

## 4. Discussion

In the present case study, we had the unique opportunity to integrate different types of disconnections emerged from different techniques that were performed on the same patient presenting with a post-surgical frontal lesion.

This integrated picture, derived *via* the adoption of a multi-modal analysis, explained patient’s behavior.

For instance, patient’s visuo-spatial impairment and constructional apraxia (46, 47) were not directly explained based on the focal lesion, while the multi-modal analysis revealed SDC/FDC and metabolic disconnection of occipital-parietal regions and the VIS/DAN alterations that well matched with these deficits (Figures 1–3).

Executive and memory impairment can be linked to lesion of SFG and fornix, though, the multi-modal approach captured a more widespread dysfunction of prefrontal-temporo-parietal (FPN/DAN) and meso-limbic structures (DMN) (48–50).

Indeed, even though the degree of the neuropsychological impairment appeared rather modest vis-à-vis the widespread functional alteration, the multi-modal approach revealed a fragile structure/functional scaffold (involving distributed networks) that was more subjected to transitory pathological modulation, as evident in the EEGs during delirium, with a spreading of delta activity associated with worsening in cognitive performance (Figure 4).

Therefore, this case study highlights the complexity and the clinical relevance of diaschisis in focal lesions at single-subject level.

Focal lesions produce remote physiological effects that are related to the disconnection of incoming/outgoing/passing WM fibers to/from the lesion. This SDC, in turn, causes remote metabolic and functional effects that have been documented using different techniques (PET, fMRI) (4). The mapping between anatomical disconnection, metabolic/functional disconnection, and dynamic changes of synchronization/activity remains to-date largely unknown due to the dearth of multimodal studies that have addressed these issues using multiple imaging modalities on the same subject (51).

Here we had the chance to study concurrently anatomical-metabolic-functional organization along with EEG measures in a patient with a frontal lesion. There were three main findings detected in the multi-modal mapping.

A first notable result was the presence of a widespread intra-hemispheric and inter-hemispheric SDC. The SDC involved tracts near the structural lesion, but it also extended to commissural fibers, long-range association pathways and cortico-subcortical pathways.

Secondly, this disconnection nicely matched the spatial pattern of glucose hypometabolism measured through the LI or qualitatively observed on the [^18^F]FDG SUVR map (e.g., bilateral mPFC).

This SDC-[^18^F]FDG PET result supports the hypothesis that metabolic changes reflect diaschisis (52) (e.g., neural disconnection due to reduction of direct connections/synaptic inputs). In contrast, alterations of local (ReHo) and remote synchronization (RSN independent components and FC within/between networks) were more widespread involving multiple networks. Hence different mechanisms may underly the broader FC-fMRI and ReHo dysfunction, such as the propagation of the effect through BOLD oscillations or through large-scale networks dynamics (53, 54).

Thirdly, the baseline EEG was abnormal both anteriorly near the lesion (delta activity) as well as posteriorly in the occipital lobe and was then subjected to similar changes (e.g., spreading of delta activity) during the episodes of delirium, likely reflecting the dynamic effects of delirium on a baseline altered structural-functional scaffold (29, 30).

## 5. Conclusion

This case study illustrates the presence and the complexity of remote effects induced by a brain lesion. An integrated multi-modal approach can capture multiple disconnection patterns induced by a focal lesion. These are relevant to explain patient’s behavior and to develop novel biomarkers of individualized treatment targeting networks’ dysfunction.

## Data availability statement

The original contributions presented in the study are included in the article/Supplementary material, further inquiries can be directed to the corresponding author.

## Ethics statement

The studies involving human participants were reviewed and approved by Local Ethical Committee University of Padova. The patients/participants provided their written informed consent to participate in this study. Written informed consent was obtained from the individual(s) for the publication of any potentially identifiable images or data included in this article.

## Author contributions

EM, ES, MB, AC, DC, AB, and MC: conception and design. EM, AC, and DC: acquisition of data. EM, ES, MB, AC, MA, DC, AB, and MC: analysis, interpretation of data, writing, review, and/or revision of the manuscript. All authors contributed to the article and approved the submitted version.

## Funding

MC was supported by FLAG-ERA JTC 2017 (grant ANR-17-HBPR-0001), MIUR–Departments of Excellence Italian Ministry of Research (MART_ECCELLENZA18_01), Fondazione Cassa di Risparmio di Padova e Rovigo (CARIPARO)–Ricerca Scientifica di Eccellenza 2018–(Grant Agreement number 55403), Ministry of Health Italy Brain connectivity measured with high-density electroencephalography: a novel neurodiagnostic tool for stroke-NEUROCONN (RF-2008-12366899), Celeghin Foundation Padova (CUP C94I20000420007), BIAL Foundation grant (No. 361/18), H2020 European School of Network Neuroscience-euSNN, H2020-SC5-2019-2 (Grant Agreement number 869505), H2020 Visionary Nature Based Actions For Heath, Wellbeing & Resilience in Cities (VARCITIES), H2020-SC5-2019-2 (Grant Agreement number 869505), and Ministry of Health Italy: Eye-movement dynamics during free viewing as biomarker for assessment of visuospatial functions and for closed-loop rehabilitation in stroke–EYEMOVINSTROKE (RF-2019-12369300).

## Conflict of interest

The authors declare that the research was conducted in the absence of any commercial or financial relationships that could be construed as a potential conflict of interest.

## Publisher’s note

All claims expressed in this article are solely those of the authors and do not necessarily represent those of their affiliated organizations, or those of the publisher, the editors and the reviewers. Any product that may be evaluated in this article, or claim that may be made by its manufacturer, is not guaranteed or endorsed by the publisher.

## Footnotes

^1^ http://www.itksnap.org/
