# Supplementary material for: Case report: Multiple disconnection patterns revealed by a multi-modal analysis explained behavior after a focal frontal damage
Source: Front Neurol. 2023 Mar 17;14:1142734. doi: 10.3389/fneur.2023.1142734 (PMC10064861; doi:10.3389/fneur.2023.1142734)

Supplementary Material

**Case report: multiple disconnection patterns revealed by a multi-modal analysis explained behavior after a focal frontal damage.**

Elena Monai^1,2^, Erica Silvestri^3,4^, Marta Bisio^4,5^, Annachiara Cagnin^1,2,4^, Marco Aiello ^6^, Diego Cecchin^4,7^, Alessandra Bertoldo^3,4^ and Maurizio Corbetta^1,2,4,8^

**Supplementary Figure 1: *Brain lesion***. Axial, coronal and sagittal views of patient’s post-surgical brain lesion.


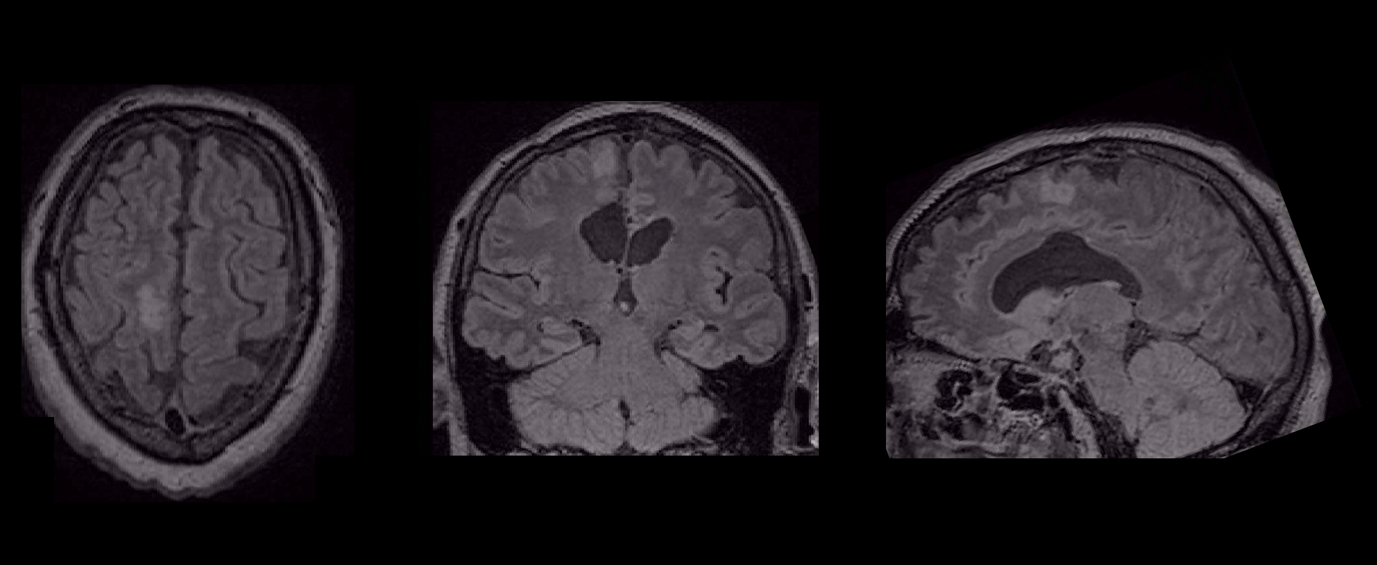


**Supplementary Figure 2: *EEG during delirium*.** EEG (standard 10-20 international system) acquired during the first episode of delirium showing tracts of bilateral slowing (selected window of 30 seconds, sampling rate 128 Hz, high-pass filtering 0.5 Hz; low-pass filtering 45 Hz; average-referenced).


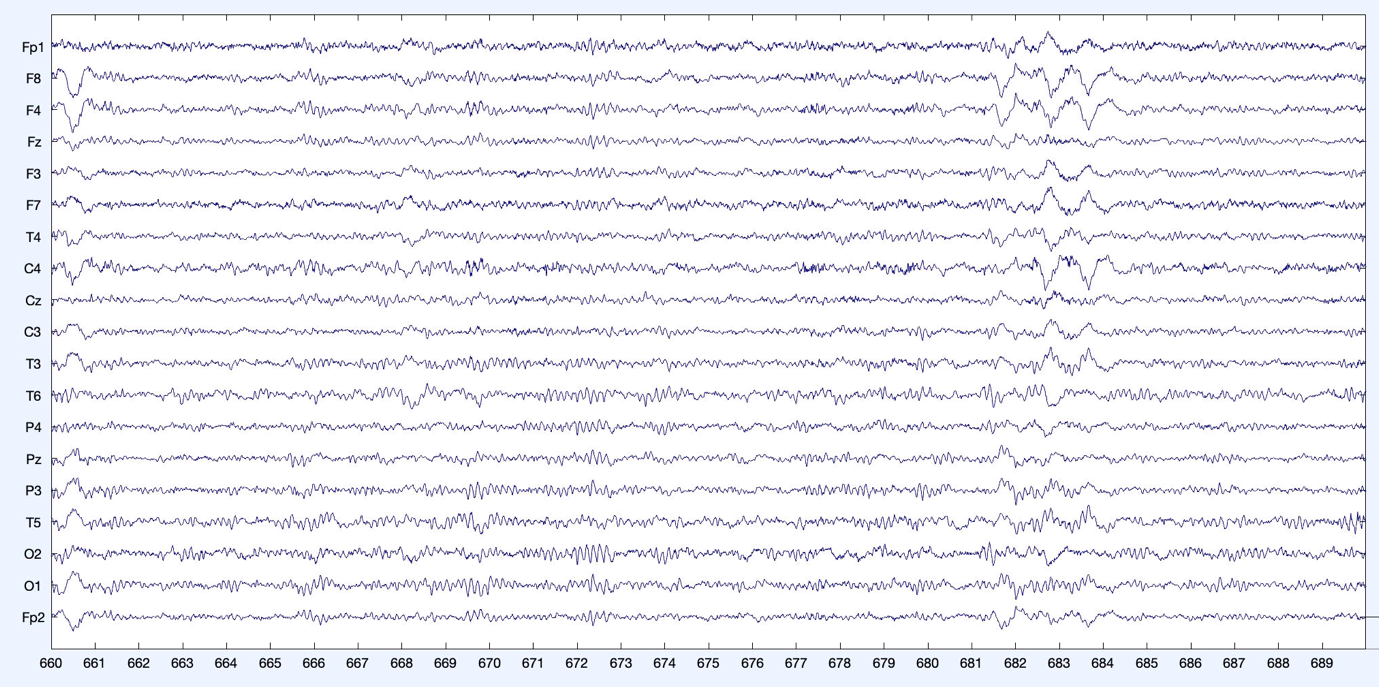


**Supplementary Figure 3: *Neuropsychological evaluations***. Neuropsychological tests performed out-of-delirium (orange) and during delirium (blue). Scores are reported as E.S. (E.S.=0 deficit; E.S.=1 borderline; E.S.=2-4 normal) in the left top radar graphic for attentional matrix, FAB, digit span, fluency and Rey Figure. Dashed lines indicate cut-off for TMT-A (66”), TMT-B (149”; during delirium >3 errors and interrupted), immediate prose memory (6), recall prose memory (9; during delirium 0/28), Clock drawing (7).


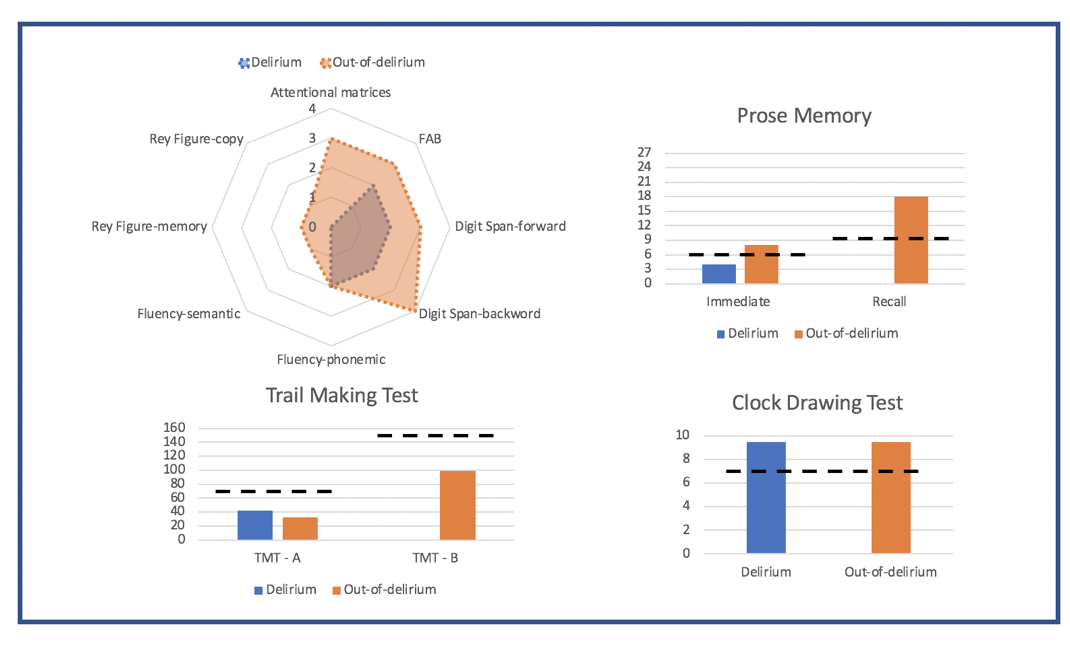


**Supplementary Figure 4: *Distribution of [^18^F]FDG SUVR Laterality Indices (LI).***

SUVR LI in the patient as compared to the distribution of SUVR LI in the healthy controls (light blue area controls average minus/plus two standard deviations) across anatomical ROIs of the Hammersmith atlas. Nearly all ROIs show a relative hypometabolism in the right hemisphere. Statistically significant regions are noted as black dots and red rectangles.


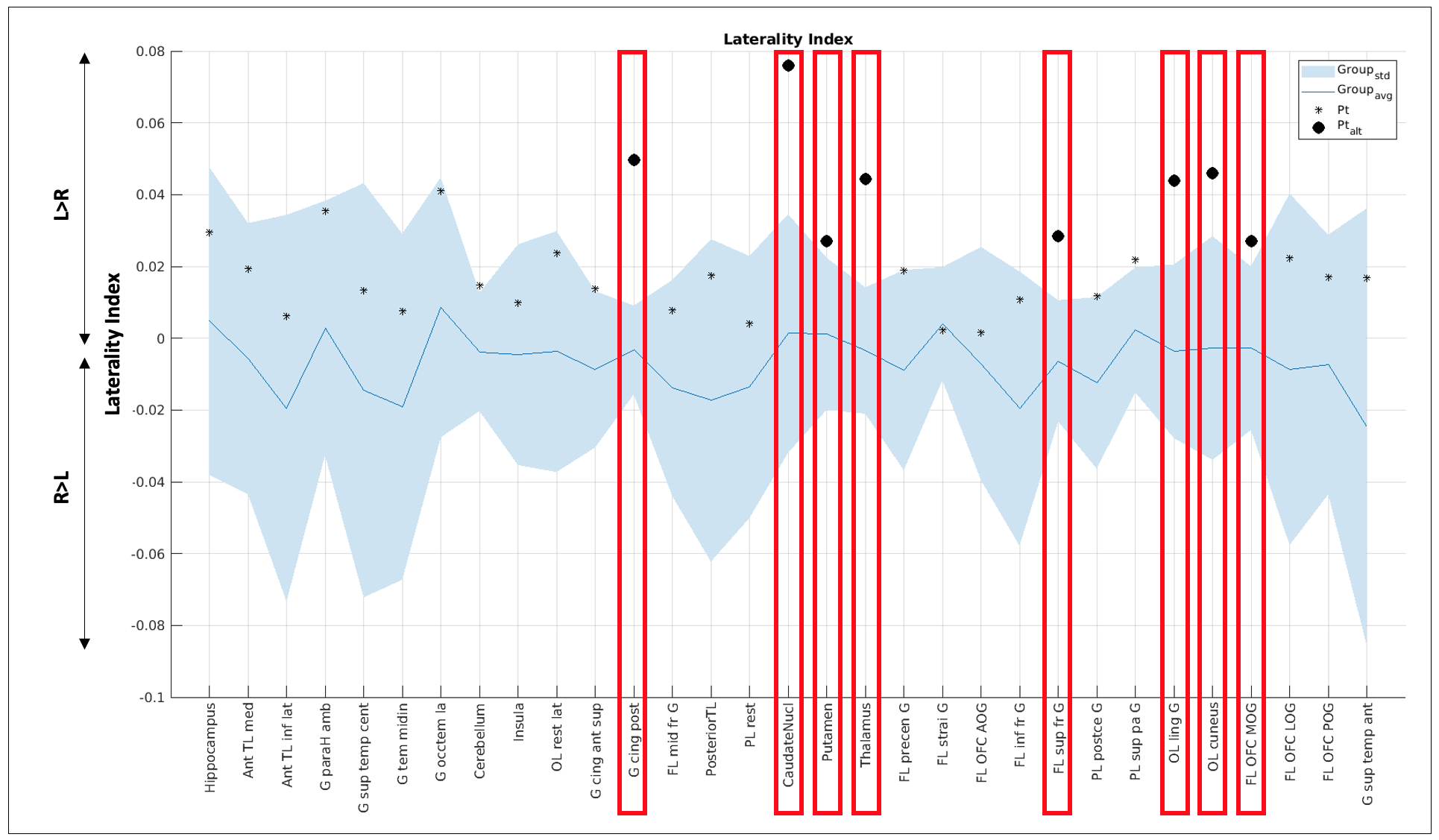


**Supplementary Figure 5: *Structural disconnection and metabolic asymmetry*.** T1-weighted structural MRI scan showing the structural disconnection without threshold (blue) and regions with significative metabolic asymmetry (>2SD) (green).

**
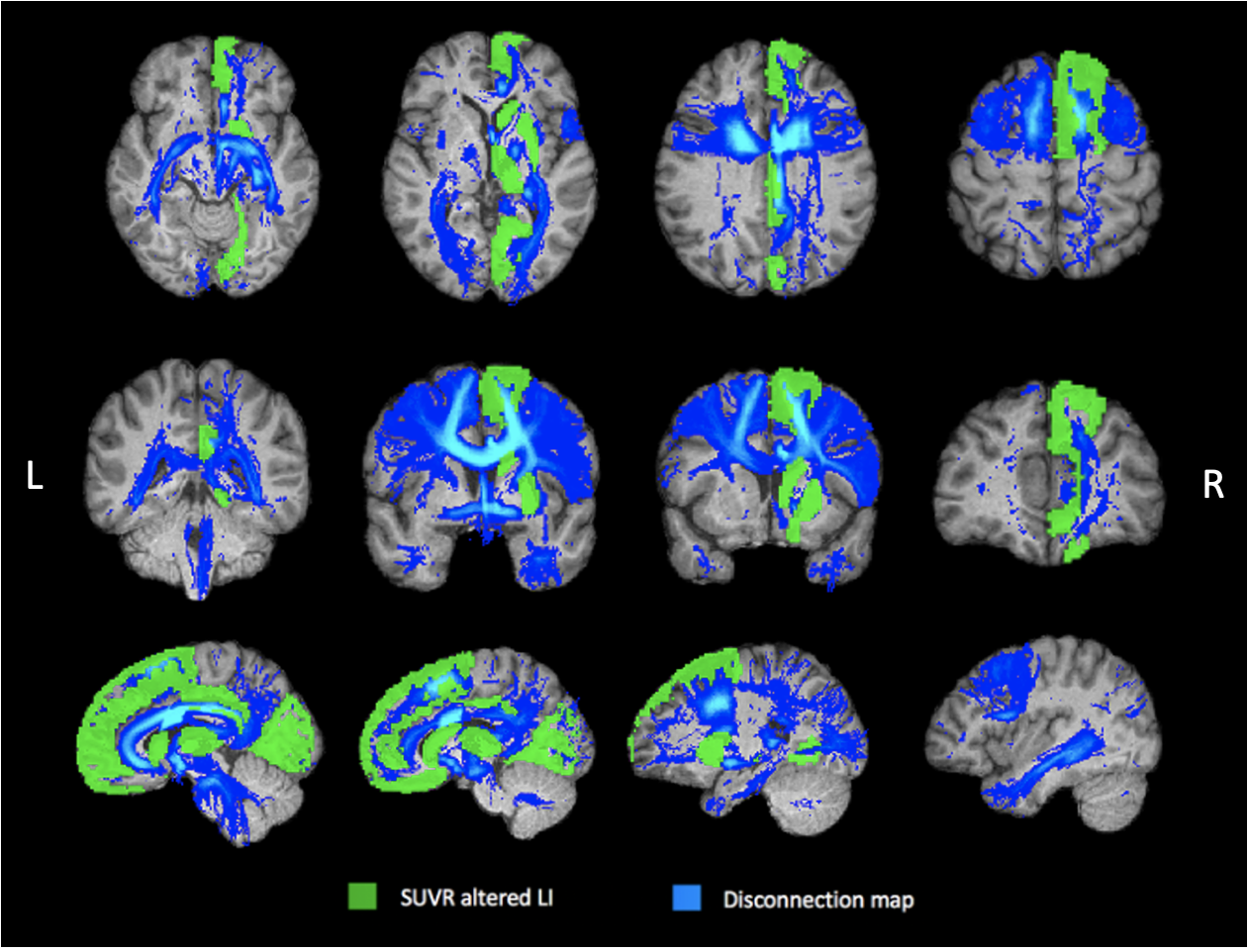
**

**Supplementary Figure 6*.*** ***[^18^F]FDG PET.*** Patient’s [^18^F]FDG PET SUVR map in the axial sections qualitatively showing an hypometabolism mainly in right ventromedial and dorsomedial prefrontal cortices (VMPFC, DMPFC) and frontal regions, subcortical structures including the thalamus (T), caudate nucleus and putamen (NC, P) and extending posteriorly to midline cortices as posterior cingulate cortex (PCC) and visual cortex (cuneus and lingual gyrus, LG). A slight right temporal and contralateral medial prefrontal hypometabolism is also visible.


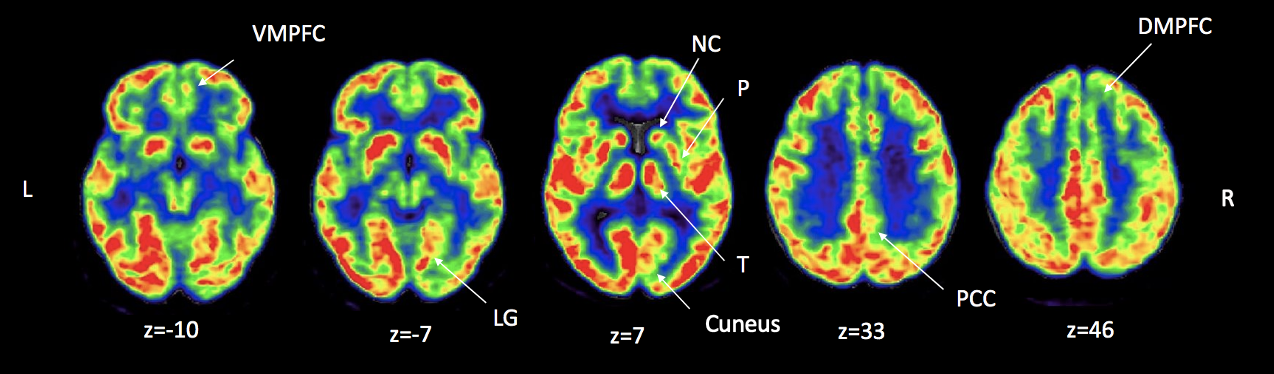


**Supplementary Figure 7:** ***Resting state networks spatial pattern alterations.***

Spatial pattern of all the independent components resulting significantly altered. Each panel shows the components within each RSN (i.e., visual, dorsal attention, fronto-parietal, default mode, cingulo-opercular, and cognitive control network). Within each panel, the spatial pattern of each component (e.g., IC31) for the group of healthy subjects (top) and the patient (bottom).


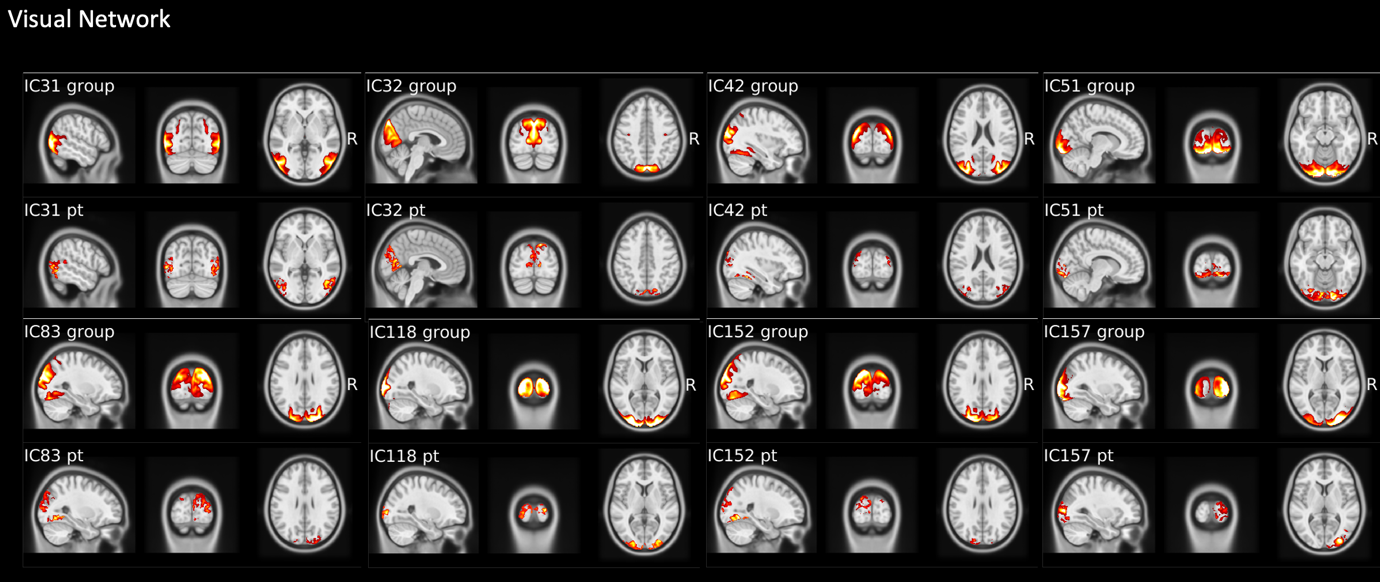


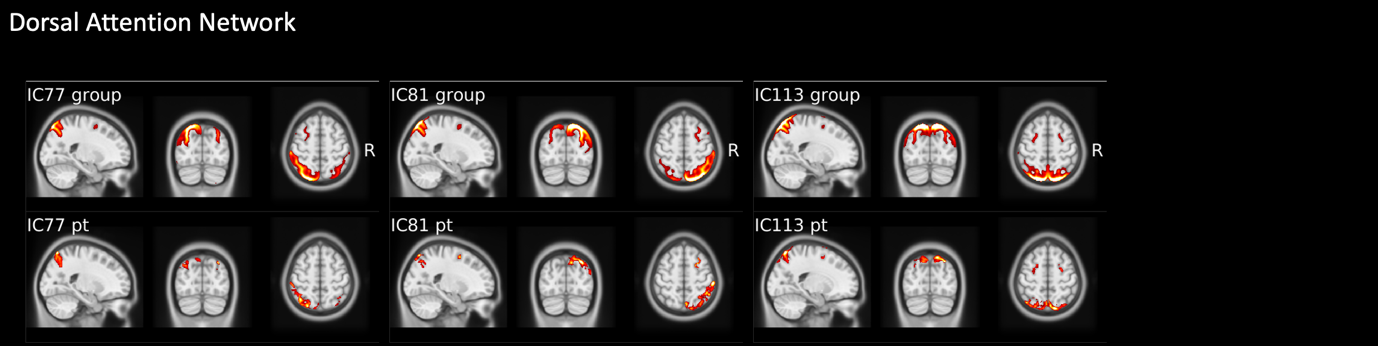


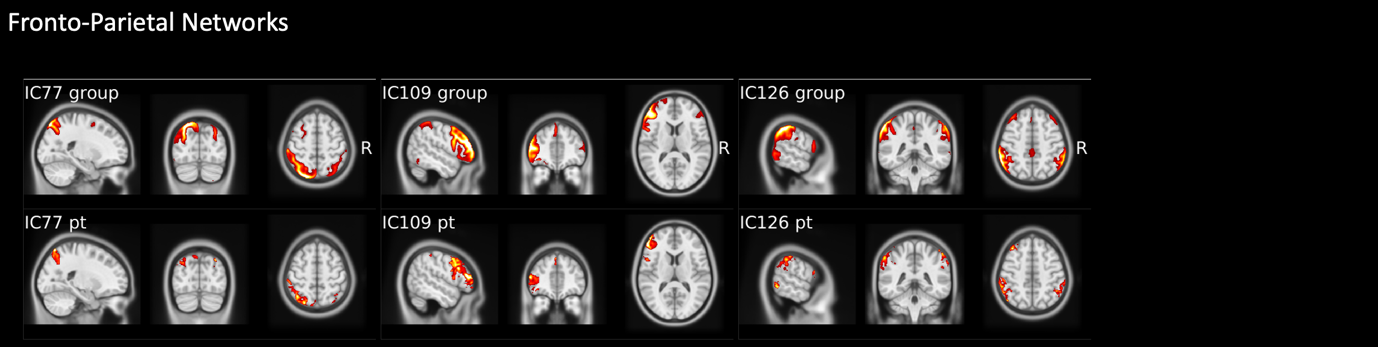


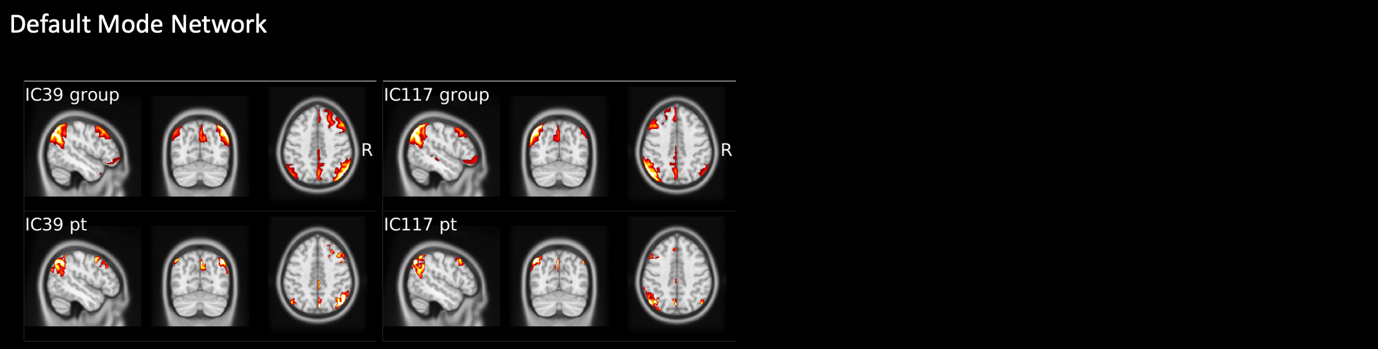


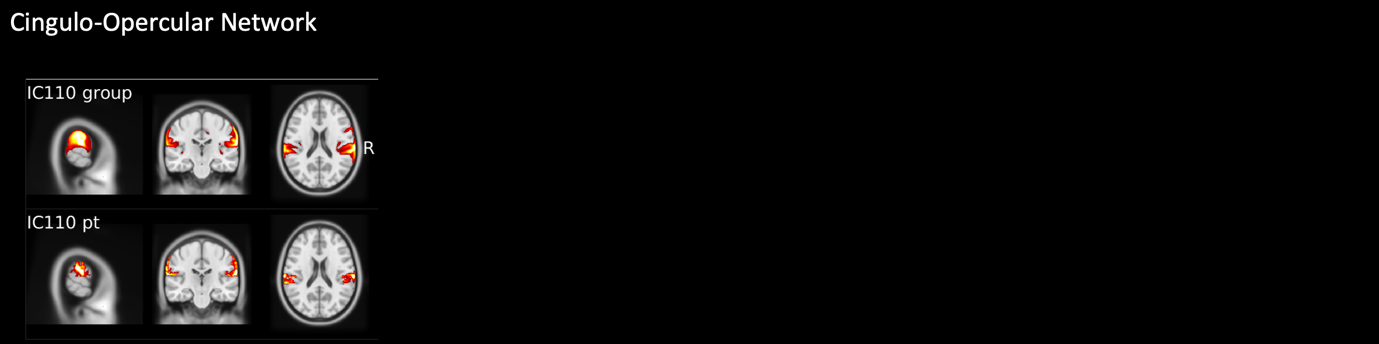


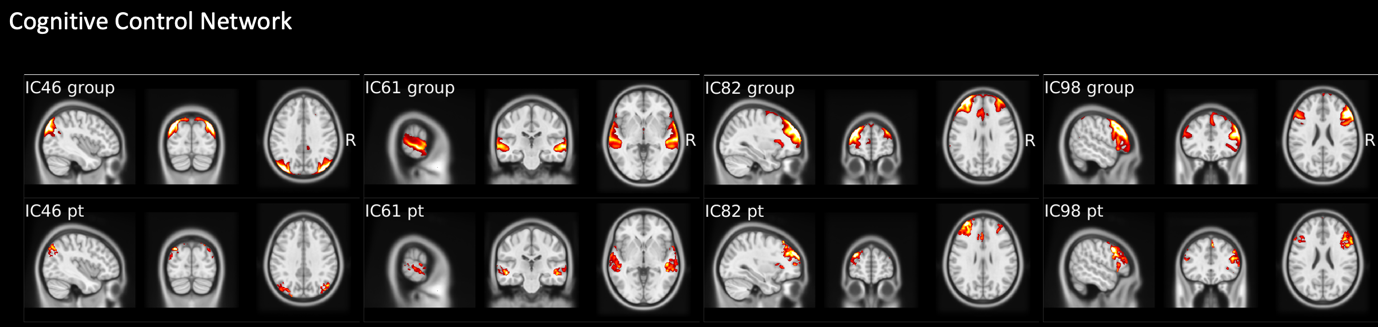


**Supplementary Figure 8:** ***Delta cosine similarity (ΔCS_σ_).*** Delta cosine similarity (ΔCS_σ_) of independent functional components of RSN computed as difference between patient’s cosine similarity (CS) and controls’ average CS and normalized for the controls’ CS standard deviation (σ). Components with ΔCS_σ_>2 were labelled as abnormal either in terms of spatial pattern or magnitude of connectivity.


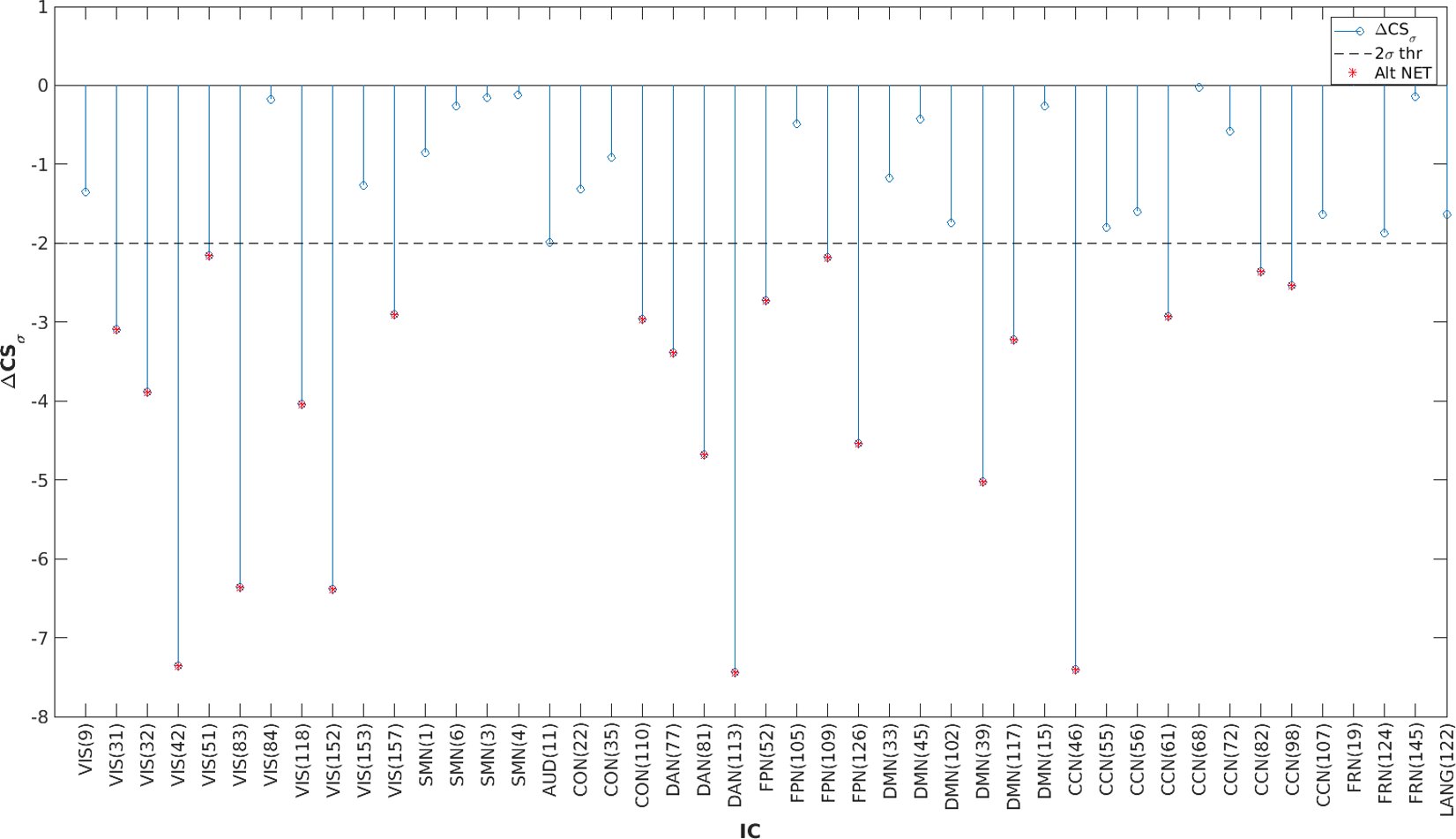


**Supplementary Figure 9:** ***Relationship between voxel-wise map of glucose hypometabolism (LI) and resting state alterations with structural disconnection (SDC).*** In pink are depicted all the voxels belonging to the resting state networks that were altered in terms of cosine similarity compared to the healthy group. In green voxels belonging to regions with altered LI. In blue SDC (threshold 0.2). To note that a voxel is marked as “functionally altered” when the independent component it belongs to shows significant alterations in the spatial pattern and/or a decrease in the connectivity strength.


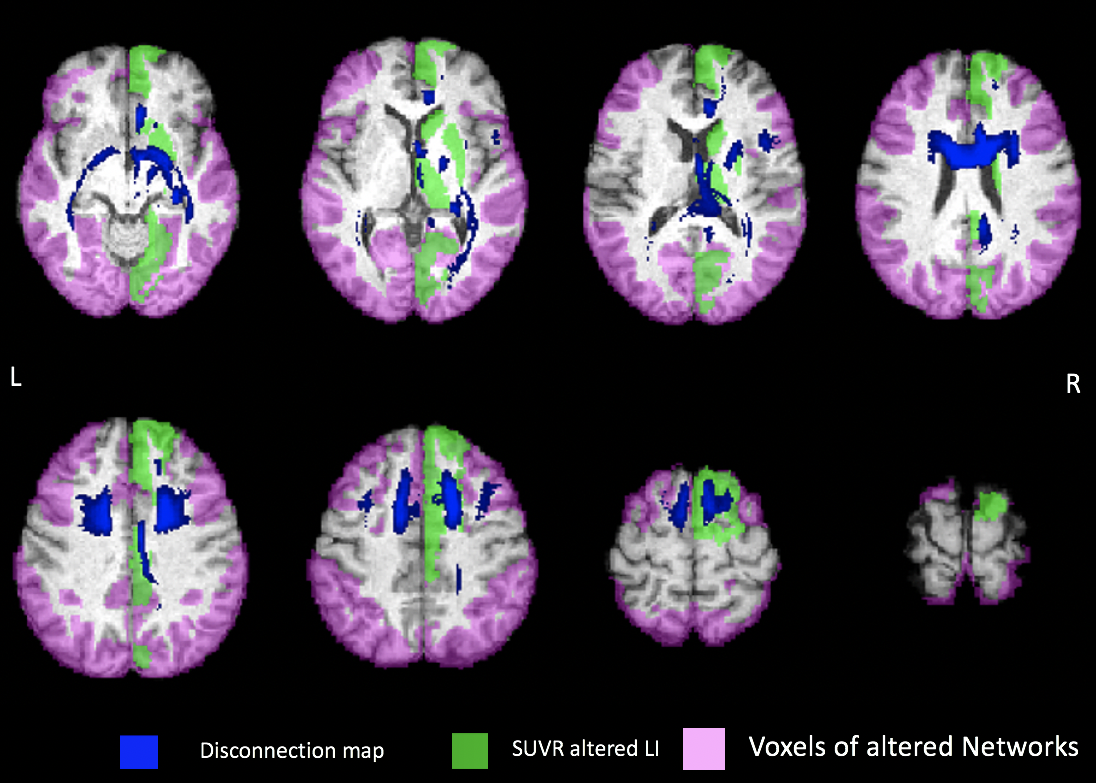


**Supplementary Table I:** **Neuropsychological tests**. Neuropsychological tests performed during delirium (11 days after admission), 24 days after admission and at 1 month after discharge. Note that 24 days after admission correspond to 4 days after the execution of the PET/fMRI scan. At 11 days after admission, during delirium, attentive matrix, TMT-B, semantic fluency, immediate and deferred prose memory (ENB = *esame neuropsicologico breve*), figure Rey (memory and copy) were deficitary. At 24 days after admission only incidental phonemic memory (z score < -2) and Rey figure (memory and copy) were deficitary. The former was deficitary at baseline condition (1 month after discharge), when the performance was also borderline on Rey figure tests, as well as in semantic fluency. During the second evaluation immediate and deferred memory were tested with Babcock story (1930) (cut-off 4.5). Scores were reported as equivalent scores (E.S.) according to a 5-point scale used to standardize NPE after adjusting for age and education (E.S.=0 deficit; E.S.=1 borderline; E.S.=2-4 normal)^43^ for attentive matrix, FAB, forward and backward digit span, phonemic and semantic fluency, Corsi tests, Rey figure tests. The other items were considered according to each cut-off (TMT-A cut-off=66”; TMT-B cut-off=149”; prose memory immediate cut-off=6; prose memory deferred cut-off=9; Clock drawing test cut-off=7) and incidental phonemic memory according to z score (deficit if z<-2).

| **TEST** | ***11 days after admission*** | ***24 days after admission*** | ***1 month after discharge*** |
| --- | --- | --- | --- |
| Attentional matrices | ES = 0 | ES=3 | ES=3 |
| Trail Making Test - A | 42" | 33" | 32" |
| Trail Making Test - B | >3 errors/interrupted | 92" | 99" |
| FAB | ES = 2 | / | ES=3 |
| Fluency-phonemic | ES = 2 | ES=2 | ES=2 |
| Fluency-semantic | ES= 0 | ES=2 | ES=1 |
| Digit Span-forward | ES=2 | ES = 4 | ES=3 |
| Digit Span-backword | ES=2 | ES = 4 | ES=4 |
| Prose Memory-immediate (ENB) | 4/28 | / | 8/28 |
| Prose Memory-deferred (ENB) | 0/28 | / | 18/28 |
| Prose memory -immediate (Babcock) | / | 6.9/8 | / |
| Prose memory-deferred (Babcock) | / | 6.6/8 | / |
| Incidental phonemic memory | / | z=-2.3 | z=-2.3 |
| Rey Figure-memory | ES= 0 | ES=0 | ES=1 |
| Rey Figure-copy | ES=0 | ES=0 | ES=1 |
| Corsi Test -direct | / | ES= 4 | ES=4 |
| Corsi Test-Indirect | / | ES= 4 | ES=4 |
| Clock drawing test | 9.5/10 | / | 9.5/10 |
| Ideomotor apraxia | ES=3 | / | / |
| Orofacial apraxia | ES =4 | / | / |

**Supplementary Table II:** **overalp between WM tracts and patient’s SDC map.** % of overlap between each tract defined in the BCB toolkit atlas (probability threshold set to 0.1) and the patient’s structural disconnection map (probability threshold set to 0.2). A tract was labeled as impaired if the percentage overlap was greater than 10% (i.e., when more than the 10% of the tract volume overlapped the structural disconnection map) (R = right, L = left).

| Tract | Dimension (#vox) | Overlap with discon (#vox) | % Overlap | altered flag |
| --- | --- | --- | --- | --- |
| Anterior Commissure | 8295 | 3319 | 40 | 1 |
| Anterior Thalamic Projections L | 102362 | 9905 | 10 | 0 |
| Anterior Thalamic Projections R | 101685 | 18171 | 18 | 1 |
| Arcuate Anterior Segment L | 23570 | 0 | 0 | 0 |
| Arcuate Anterior Segment R | 39520 | 2771 | 7 | 0 |
| Arcuate Long Segment L | 29261 | 0 | 0 | 0 |
| Arcuate Long Segment R | 13413 | 636 | 5 | 0 |
| Arcuate Posterior Segment L | 32945 | 0 | 0 | 0 |
| Arcuate Posterior Segment R | 43079 | 171 | 0 | 0 |
| Cingulum L | 69111 | 3152 | 5 | 0 |
| Cingulum L anterior | 55629 | 2908 | 5 | 0 |
| Cingulum L posterior | 13784 | 1 | 0 | 0 |
| Cingulum R | 45266 | 10515 | 23 | 1 |
| Cingulum R Anterior | 45821 | 10816 | 24 | 1 |
| Cingulum R Posterior | 12688 | 1556 | 12 | 1 |
| Corpus callosum | 813854 | 69844 | 9 | 0 |
| Cortico Spinal L | 43740 | 5993 | 14 | 1 |
| Cortico Spinal R | 59063 | 15760 | 27 | 1 |
| Face U tract L | 2535 | 0 | 0 | 0 |
| Face U tract R | 2947 | 0 | 0 | 0 |
| Fornix | 24940 | 6112 | 25 | 1 |
| Frontal Aslant Tract L | 48867 | 9615 | 20 | 1 |
| Frontal Aslant tract R | 46436 | 16983 | 37 | 1 |
| Frontal Commissural | 330551 | 46550 | 14 | 1 |
| Frontal Inferior longitudinal L | 12878 | 198 | 2 | 0 |
| Frontal Inferior longitudinal R | 20790 | 3755 | 18 | 1 |
| Frontal Orbito Polar L | 11428 | 0 | 0 | 0 |
| Frontal Orbito Polar R | 13288 | 43 | 0 | 0 |
| Frontal Superior Longitudinal L | 38915 | 3649 | 9 | 0 |
| Frontal Superior Longitudinal R | 38296 | 7231 | 19 | 1 |
| Fronto Insular tract1 L | 0 | 0 | - | 0 |
| Fronto Insular tract1 R | 0 | 0 | - | 0 |
| Fronto Insular tract2 L | 0 | 0 | - | 0 |
| Fronto Insular tract2 R | 2303 | 0 | 0 | 0 |
| Fronto Insular tract3 L | 2089 | 0 | 0 | 0 |
| Fronto Insular tract3 R | 9256 | 1313 | 14 | 1 |
| Fronto Insular tract4 L | 6903 | 1 | 0 | 0 |
| Fronto Insular tract4 R | 8067 | 668 | 8 | 0 |
| Fronto Insular tract5 L | 4673 | 0 | 0 | 0 |
| Fronto Insular tract5 R | 9115 | 21 | 0 | 0 |
| Fronto Marginal tract L | 9784 | 0 | 0 | 0 |
| Fronto Marginal tract R | 16162 | 92 | 1 | 0 |
| Fronto Striatal L | 115797 | 8739 | 8 | 0 |
| Fronto Striatal R | 110784 | 16983 | 15 | 1 |
| Hand-inf U tract L | 11857 | 0 | 0 | 0 |
| Hand-inf U tract R | 9761 | 0 | 0 | 0 |
| Hand-mid U tract L | 0 | 0 | - | 0 |
| Hand-mid U tract R | 5085 | 0 | 0 | 0 |
| Hand-sup U tract L | 9785 | 0 | 0 | 0 |
| Hand-sup U tract R | 8452 | 0 | 0 | 0 |
| Inferior Fronto Occipital fasciculus L | 78662 | 1392 | 2 | 0 |
| Inferior Fronto Occipital fasciculus R | 71336 | 6030 | 8 | 0 |
| Inferior Longitudinal L | 102312 | 2262 | 2 | 0 |
| Inferior Longitudinal R | 107016 | 7296 | 7 | 0 |
| Optic Radiations L | 28068 | 1720 | 6 | 0 |
| Optic Radiations R | 17353 | 4114 | 24 | 1 |
| Paracentral U tract L | 803 | 0 | 0 | 0 |
| Paracentral U tract R | 227 | 0 | 0 | 0 |
| Pons L | 66425 | 7272 | 11 | 1 |
| Pons R | 55280 | 14714 | 27 | 1 |
| Superior Longitudinal Fasciculus III L | 56917 | 73 | 0 | 0 |
| Superior Longitudinal Fasciculus III R | 95463 | 3901 | 4 | 0 |
| Superior Longitudinal Fasciculus II L | 86714 | 4457 | 5 | 0 |
| Superior Longitudinal Fasciculus II R | 93621 | 8295 | 9 | 0 |
| Superior Longitudinal Fasciculus I L | 85634 | 9295 | 11 | 1 |
| Superior Longitudinal Fasciculus I R | 76730 | 10726 | 14 | 1 |
| Uncinate L | 28385 | 0 | 0 | 0 |
| Uncinate R | 21103 | 861 | 4 | 0 |

**Supplementary Table III: Altered connections within and between RSNs.**

Left: Significantly altered pairwise FC between individual components divided by networks. The components in bold also show spatial alterations based on the DICE similarity analysis. Altered FC between RSN independent components Middle: number and percentage of pairwise FC within and between RSNs; Right: number and percentage of pairwise FC between individual components within and between RSNs in matrix form.


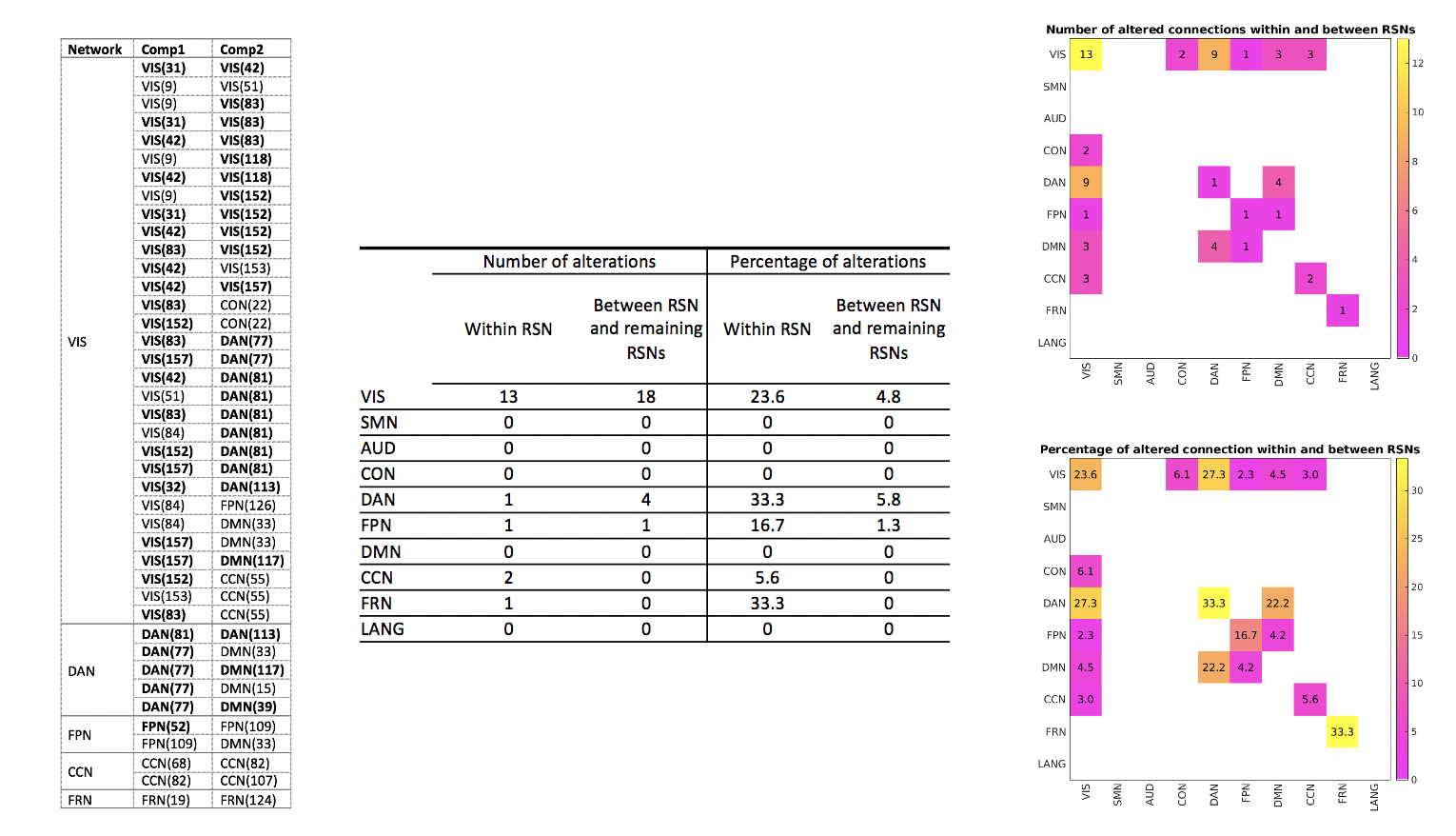

Supplement: Supplementary file 1 [file Data_Sheet_1.docx]
